# What is this very big skin lesion?

**DOI:** 10.1002/ccr3.1031

**Published:** 2017-08-01

**Authors:** Angelo Guttadauro, Silvia Frassani, Matteo Maternini, Barbara Rubino, Elena Guanziroli, Francesco Gabrielli

**Affiliations:** ^1^ Surgery Department Istituti Clinici Zucchi University of Milano‐Bicocca Monza Italy; ^2^ Surgery Department Istituti Clinici Zucchi Monza Italy; ^3^ Anatomo‐patological Department Policlinico San Donato Milano Italy; ^4^ Dermatology Department Ospedale Maggiore di Milano Univesity of Milano Milano Italy

**Keywords:** Basal cell carcinoma, distant metastasis, radiation therapy, skin lesion

## Abstract

This clinical image shows the importance of the early diagnosis and treatment of any suspicious skin lesion.

A 58‐year‐old Caucasian female was referred to our institution for anemia (hemoglobin 52 g/L) due to the bleeding of an exophytic, irregular, thick, ulcerated lesion on the left anterior chest wall. The lesion had been growing for about 10 years and had originated from a preexisting skin lesion. The rest of her past medical history was unremarkable.

We performed an incisional biopsy of the lesion. The histological findings were consistent with a metatypical basal cell carcinoma. A chest and abdomen CT scan, performed for the purpose of staging, showed a mass infiltrating the subcutaneous tissues and the intercostal left muscles with a node of 13 × 9 mm placed deeply in the lower third of the left mammary gland. No distant metastasis was observed. The patient started neoadjuvant cytoreductive radiation therapy (50 Gy in 25 fractions), and after 25 days of treatment, we obtained a significant reduction in the lesion size and the extent of bleeding.

Four weeks after the end of radiation therapy, we performed a surgical removal of the chest lesion, including the fascial layer and the lower inner quadrant of the left mammary gland, with subsequent reconstruction. Definitive histological examination confirmed an infiltrating sclerosing basal cell carcinoma with resection margin free of tumor.

The patient was discharged on the third postoperative day. Currently, she is surgically healed with no signs of local recurrence or metastasis (Figs 1 and 2).

**Figure 1:**
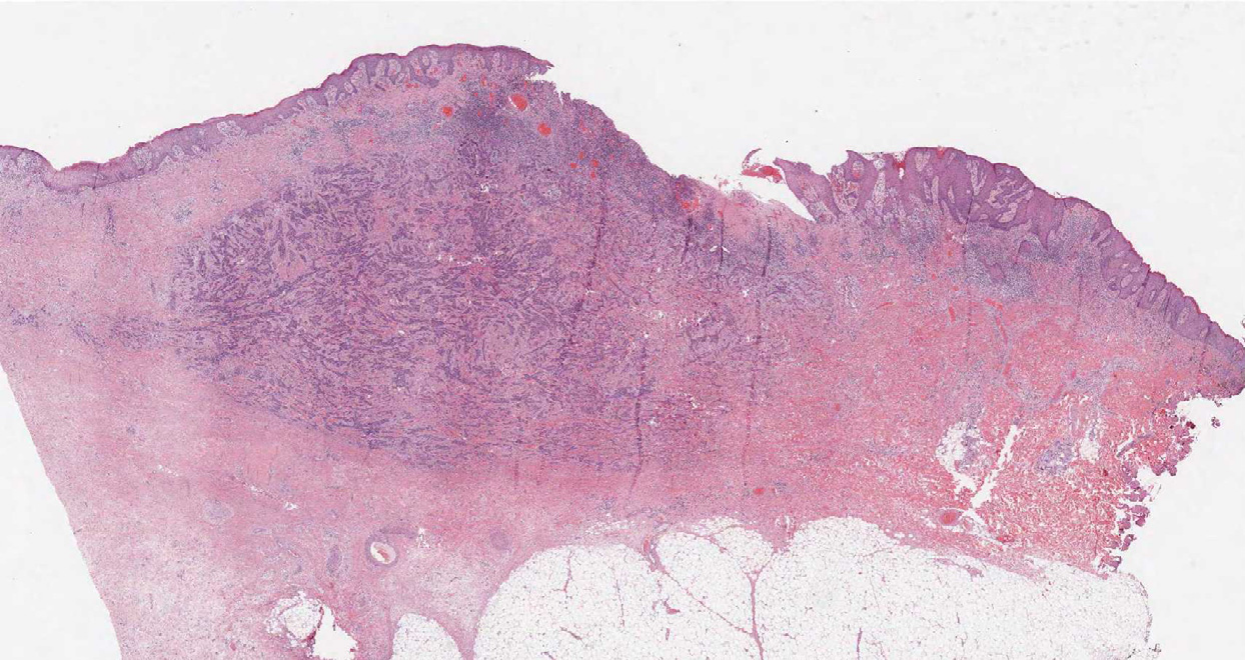
Overall view of skin and subcutaneos tissue.

**Figure 2:**
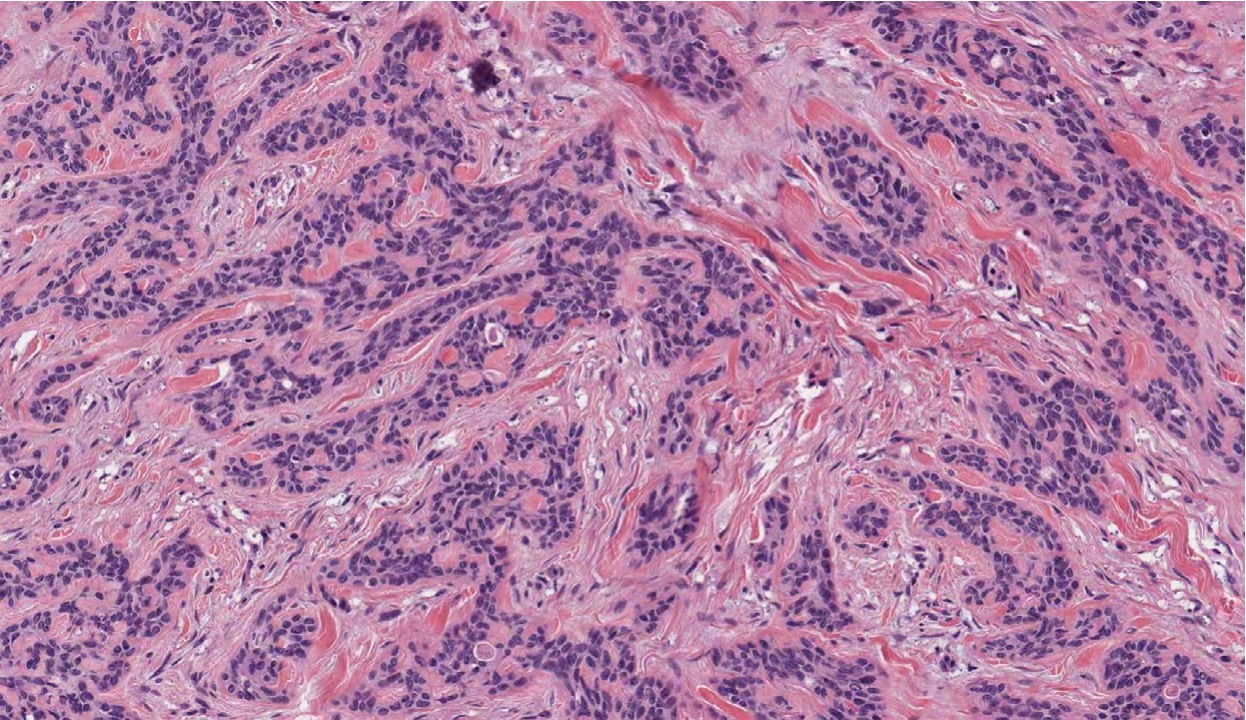
20X Basal and par‐basal elements proliferation.

## Authorship

AG, SF, and MM: Substantially contributed to conception and design, or acquisition of data, or analysis and interpretation of data; AG, MM, BR, and EG: Drafted the article or revised it critically for important intellectual content; EG and FG: Finally approved of the version to be published.

## Conflict of Interest

None declared.

